# A method to improve cotton fiber length measurement for laboratory analysis

**DOI:** 10.1016/j.mex.2020.100859

**Published:** 2020-03-12

**Authors:** Joao Morais, Jacob James, Zach Hinds, Wayne Smith, Brendan Kelly, Eric Hequet

**Affiliations:** a Texas Tech University, USA; b Texas A&M Agrilife Research, USA; c Embrapa Algodão, Brazil

**Keywords:** Ginning, Fiber length distribution, Lint cleaning, Trash, Processing

## Abstract

Cotton fiber length is an essential parameter for the cotton industry and cotton research. However, differences between industry- and laboratory-scale ginning may lead to inconsistencies between research and industry results for measured length. Seedcotton from farms is processed in large industry-scale gins, while researchers typically use small laboratory-scale gins. The proposed method successfully reduces the differences in fiber length parameters between these two types of ginning. Only one new step is needed before assessing fiber quality in lint from a laboratory-scale gin to simulate the processing effect of an industry-scale gin.

• Cotton seeds and lint are separated from seedcotton with a laboratory-scale gin.
• Lint is post-processed with a laboratory-scale lint cleaner, the micro dust and trash analyzer 3.
• The length fiber quality profile resembles the results of industry-scale ginned samples.

## Graphical abstract

**Figure:**
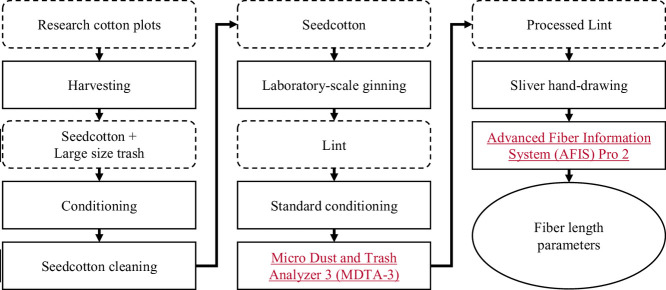

**Table:** Specifications Table

Subject Area	Agricultural and Biological Sciences
More specific subject area:	*Cotton fiber quality*
Method name:	*Post-processing of laboratory-scale cotton fiber for length measurement*
Name and reference of original method	*W.S. Anthony, W.D. Mayfield, Cotton ginners handbook, United States Department of Agriculture, Springfield.*
Resource availability	*Not applicable*

## Background

Length and length uniformity index are fiber quality parameters used in marketing cotton [1]. These two parameters are the most important properties for ring spinning, the spinning technology with the greatest market share in the world [2], [3], [4] and they are affected by genetics, environment, and processing conditions [5,6]. Spinners use these length parameters to configure their machines and predict yarn properties using the information provided with each purchased bale of cotton lint.

Farmers process their harvested upland cotton in industry-scale saw gins. Many improvements have happened in this industry since the saw gin was invented by Eli Whitney [7,8]. A typical modern industry-scale gin is composed of many parts to process tons of cotton fiber per hour, including pre and post ginning cleaning processes not proposed in the original patent [9]. Seedcotton cleaners eliminate many sticks, burrs, leaves, and some dust that is harvested with the cotton before cotton goes to the gin stand. At the gin stand, saws separate lint and cottonseed. Ginned lint is processed with lint cleaners to mechanically remove part of the non-lint content, including plant particulates called trash that have made it through the pre-cleaning and ginning process, before the fibers are pressed into bales [5,10].

Industry-scale gins require hundreds of pounds of seedcotton to function correctly, making industry-scale ginning impractical in most research applications. For example, if cotton breeders are selecting individual plants, they cannot process the harvest from one plant with an industry-scale gin. They must use laboratory-scale gins. Laboratory-scale gins are typically very basic gin saws and brushes, minimized for small scale use. These gins typically have no pre-cleaning or lint cleaning and lack uniform feed controls that industry-scale gins have. Another difference between laboratory- and industry-scale gins is the amount of used power. An industry-scale gin operates at 0.38–0.82 kW/saw, while a laboratory-scale may run at 0.04 kW/saw [9,11].

Fibers can break when aggressive mechanical forces are applied during the cleaning processes used in the ginning industry. Factors such as the smaller throughput, slower and less consistent feed speeds, fewer cleaning stages, and lower levels of power applied to process the fibers during laboratory-scale ginning may affect the fiber quality profile. This results in differences within the length profile of a sample that was processed in an industry-scale gin. If these differences are statistically significant, decisions taken on data from laboratory-scale gins may not reflect real-world performance of a particular cotton sample in an industry-scale gin. However, if additional treatment could be applied to laboratory-scale ginned fibers, the differences between the two ginning methods may be reduced. The objective of the method described in this research is to identify an instrument that can be used after laboratory-scale ginning to reduce the differences in the fiber length quality profiles between laboratory- and industry-scale ginning.

## Method

### Sample preparation

1. Harvest seedcotton by hand or using a cotton stripper harvester.
2. Store the harvest at the desired atmospheric conditions, such as 21 ± 1 °C and relative humidity of 55 ± 5% for at least seven days after which equilibrium with the ambient conditions is assumed. This will minimize the risk to introduce environmental variation not related to the field experiment.
3. If stripper-harvested, clean the seedcotton to remove large sticks and burrs that can damage or hinder the laboratory-scale gin.
4. Feed a laboratory-scale saw-gin between 10 and 20 saws with 4–6 g of seedcotton per saw per minute. This feed rate has been found to be optimal for the processing of samples in an efficient and consistent manner.
5. Condition the lint at standard atmospheric conditions for cotton fiber classification and testing, 21 ± 1 °C and relative humidity of 65 ± 2% [12] for at least two days after which equilibrium with the ambient conditions is assumed.
6. Verify the settings of the micro dust and trash analyzer 3 (MDTA 3). If necessary, adjust the gauges between the feed roller and feed plate, the feed plate and opening roller, and the opening roller and trash knife to 0.1 mm.
7. Turn on the MDTA 3 and adjust the vacuum to 3.5 mbar in the fiber channel and 2.5 mbar in the dust channel.
8. Evenly spread 4–5 g of conditioned lint on the whole surface of the conveyor belt of the MDTA 3 and press the fibers at the end of the feed belt with the feed roller.
9. Close the rotor box and feed the lint sample into the machine.
10. Recover the sliver of processed fibers from the rotor ring box and spread it evenly on a flat surface.
11. Sample small portions of processed lint from several parts of the sliver up to 0.5 g.
12. Repeat steps 8–11 for each laboratory replication from each sample that will be tested for fiber length quality.
13. Use each sampled mass of cleaned lint to hand-draw a uniform sliver of fibers with a length of 25–30 cm.
14. Feed the slivers to an Advanced Fiber Information System (AFIS) Pro 2 testing 3000 fibers per sliver.
15. Retrieve the report.

### Comparison with other laboratory instruments and validation of the method

The objective of the proposed method is to help cotton researchers to simulate the fiber length quality profile of cotton ginned in an industry-scale gin with a laboratory-scale gin. This would prevent researchers from making conclusions that cannot be reproduced under industrial conditions.

The MDTA 3 is not the only available laboratory-scale lint cleaner. Shirley analyzers from the Shirley Institute and SDL Atlas are tools frequently used to determine the trash in cotton samples [13] that could also be used to post-process lint.

To evaluate the proposed method, we performed an experiment comparing the fiber length quality profile of cotton samples ginned in an industry-scale gin with samples ginned in a laboratory-scale gin, with and without post-processing with different types of laboratory-scale lint cleaners.

### Plant material

A set of 20 entries with a wide range of fiber length properties was used (Table S1). These entries were planted at Texas Tech Research Farm, Lubbock, TX, in crop year 2018. The double-row plots were 6.1 m long, with a density of 10 seeds per meter to simulate regional commercial planting practices. The seeds were planted on loam soil. Drip irrigation was applied, and the recommendations for irrigated cotton production in the region were followed [14]. During the experiment, the accumulated rainfall was 272 mm and the growing degree days (GDD_15.6_) were 1308 units [15].

Each plot was harvested into mesh bags with a John Deere stripper harvester with no field cleaner, resulting in seedcotton samples with a high amount of sticks and burrs. The seedcotton was conditioned for seven days at 21 ± 1 °C and relative humidity of 55 ± 5%.

### Cleaning and ginning

After bringing all seedcotton samples to the same atmospheric conditions, lint from each sample was processed with five different methods. Initially, each sample was divided into two subsamples. One subsample was passed through the seedcotton cleaners, gin stand, and lint cleaners of an industry-scale Imperial III Lummus microgin to obtain a representative industry-scale lint sample (Reference method). Another subsample was passed through the seedcotton cleaners of the Imperial III Lummus microgin and recovered before the gin stand. The cleaned seedcotton was then ginned with a tabletop laboratory-scale gin (Dennis Manufacturer, Athens, TX) to obtain a representative laboratory-scale lint sample.

**Fig. 1:**
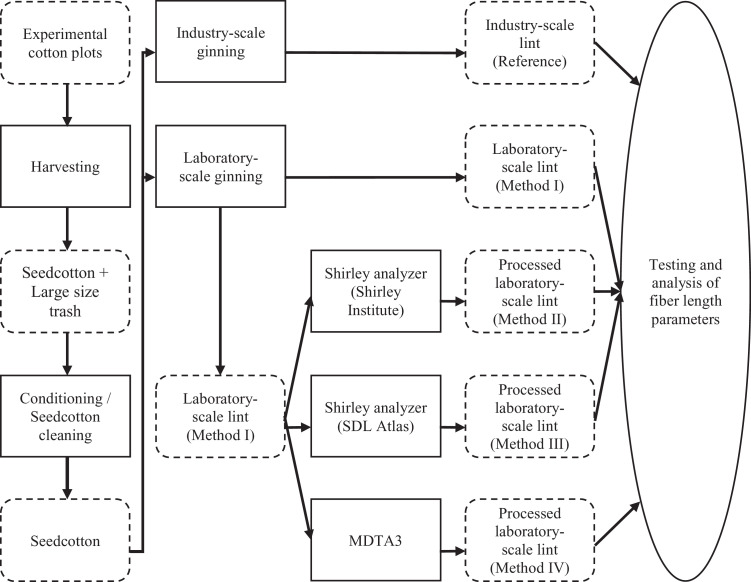
Flowchart of the validation method.

The laboratory-scale lint was divided into four subsamples of 100 g. The first subsample was sent to analysis (Method I). The second subsample was processed through a Shirley analyzer from the Shirley Institute (Method II) (Figure S1A). The third subsample was passed through a Shirley analyzer MK2 from SDL Atlas (Method III) (Figure S1B). Both Shirley analyzers were set up following the standard protocol for non-lint content measurement [13]. The fourth subsample was cleaned with an MDTA 3 from Suessen (Method IV) (Figure S1C) (Fig. 1).

### Fiber quality testing

We used an Advanced Fiber Information System (AFIS) Pro 2 (Uster) with five laboratory replications of 3000 fibers per sample to assess the length fiber quality profile of the 20 entries and five types of lint per entry. The AFIS is a sensitive instrument used to analyze fiber length parameters of individual fibers [14,16]. The AFIS has a pinned cylinder that individualizes fibers and trash. It creates fiber length distributions with 40 bins of 1.27 mm after testing 3000 fibers. Both the entire length distribution and length parameters extracted from the distribution, such as short fiber content by number (SFCn, fibers with length ≤ 12.7 mm), mean length (Ln), and length of the 5% longer fibers by number (L5%) are reported by this instrument [17,18]. Statistical differences were measured using a paired *t*-test at 95% of confidence.

### Differences for trash and neps counts

Statistical differences are observed among the treatments for trash and neps (entanglements of fibers) (Table 1). Lint from laboratory-scale ginning (Method I) exhibits a smaller number of neps and a higher content of trash, with greater values for visible foreign matter, trash per gram, and dust per gram. The presence of a lint cleaner in the industry-scale ginning (Reference method) may be a reason for these differences [19]. Indeed, it is well documented that aggressive mechanical handling from industrial lint cleaners not only removes trash particles but also tends to create fiber entanglements, i.e., neps, entanglements of fibers that reduce yarn spinning efficiency and yarn quality [20,21].

**Table 1:** Average values of trash and neps parameters in a dataset of 20 genetic materials, processed with five different ginning approaches.

Lint type	Neps per gram (count)	Visible Foreign Matter (%)	Trash per gram (count)	Dust per gram (count)
Reference method	353	3.11	156	427
Method I	196*	3.71*	185*	501*
Method II	256*^,^^a^	0.28*	16*	50*
Method III	257*^,^^a^	0.33*	21*	49*
Method IV	258*^,^^a^	0.78*	43*	128*

⁎ Treatments statistically different from industry-scale lint at 5% of significance.

a Treatments statistically different from laboratory-scale lint at 5% of significance. Reference method: industry-scale ginning; Method I: laboratory-scale ginning; Method II: laboratory-scale ginning + Shirley analyzer from Shirley Institute; Method III: laboratory-scale ginning + Shirley analyzer MK2; Method IV: laboratory-scale ginning + MDTA-3 from Suessen.

Applying a laboratory-scale lint cleaner to the lint from the regular laboratory-scale gin reduces the trash content in the samples and increases the number of neps. The numbers of neps are statistically different from the regular laboratory-scale lint, but they are still lower than the industry-scale lint (Table 1).

The differences are indications that although additional processing simulates industrial processing, it is not a perfect replacement. The next step in the research was to determine how each laboratory-scale lint cleaner altered fiber length parameters and distributions in the samples.

### Differences for fiber length parameters

There are statistical differences between industry- (Reference method) and laboratory-scale (Method I) lint samples for short fiber content by number (SFCn), which is defined as fibers less than or equal to 12.7 mm, and mean length by number (Ln) (Table 2). However, longer fibers as represented by the average length of the 5% longest fibers in the measured sample (L5%), are not statistically different between these samples [16]. Cotton samples generated by Method I represent a common scenario in cotton fiber length research. Nevertheless, these samples are different from the Reference method, the expected outcome in the cotton ginning industry.

**Table 2:** Average values of fiber length parameters in a dataset of 20 genetic materials with a wide range of length properties, processed with five different ginning approaches.

Lint type	Short fiber content by number (%)	Mean length by number (mm)	5% longest fibers by number (mm)
Reference method	30.3	18.8	34.0
Method I	26.8*	19.6*	34.3
Method II	27.8*	19.3*	34.3
Method III	32.3*	18.0*	33.5*
Method IV	29.2	19.0	34.0

⁎ Treatments statistically different from industry-scale lint at 5% of significance. Reference method: industry-scale ginning; Method I: laboratory-scale ginning; Method II: laboratory-scale ginning + Shirley analyzer from Shirley Institute; Method III: laboratory-scale ginning + Shirley analyzer MK2; Method IV: laboratory-scale ginning + MDTA-3 from Suessen.

While the Shirley analyzer from the Shirley Institute (Method II) increases the SFCn and decreases the Ln, it is not enough to eliminate the statistical difference with the industry-scale lint type. When this instrument processes a sample, it may break and remove some fibers from the sample, altering the measured length parameters.

Lint processed with the Shirley analyzer MK2 (Method III) has a different fiber quality profile in comparison with the lint from industry-scale ginning for all the three analyzed parameters. This machine was developed to perform the same processing as the Shirley analyzer from the Shirley Institute. Nevertheless, there are design differences between both instruments (Figures S1A and S1B). The Shirley analyzer MK2 increases sample SFCn and reduces Ln and L5% in comparison to industry-scale lint type samples. Fibers are likely to be broken and kept in the sample, increasing the SFCn. Since the overall fiber length was reduced, L5% and Ln were decreased.

Processing industry-scale lint type with the MDTA-3 (Method IV) (Figure S1C) eliminates the statistical differences between industry- and laboratory-scale lint types among the samples in the dataset. This treatment is the proposed method, and it could be used as a proxy to simulate the fiber length profile from industry-scale ginning.

The MDTA 3 is a versatile instrument with many applications related to cotton testing. Originally created to evaluate dust, trash, and fiber fragments in a cotton sample [22], the MDTA 3 is currently used to monitor the cleaning efficiency of lint cleaners, evaluate the quantity and type of trash in cotton lint samples at commercial gins [23,24], quantify the trash content in slivers at a spinning mill [25], determine the blending efficiency between cotton and synthetic fibers [26], and produce a sliver for laboratory-scale spinning by the Quickspin system [27].

We propose the MDTA 3 can be used to overcome the differences between industry- and laboratory-scale ginning imparted by mechanical processing. Cotton breeders can post-process their samples from laboratory-scale gins and simulate the processing effect of samples ginned in industry-scale gins. Therefore, breeders can make selection decisions that reflect the fiber length profile that the samples may present under commercial conditions, the condition under which their released varieties will ultimately be judged.

### Differences for fiber length distributions

**Fig. 2:**
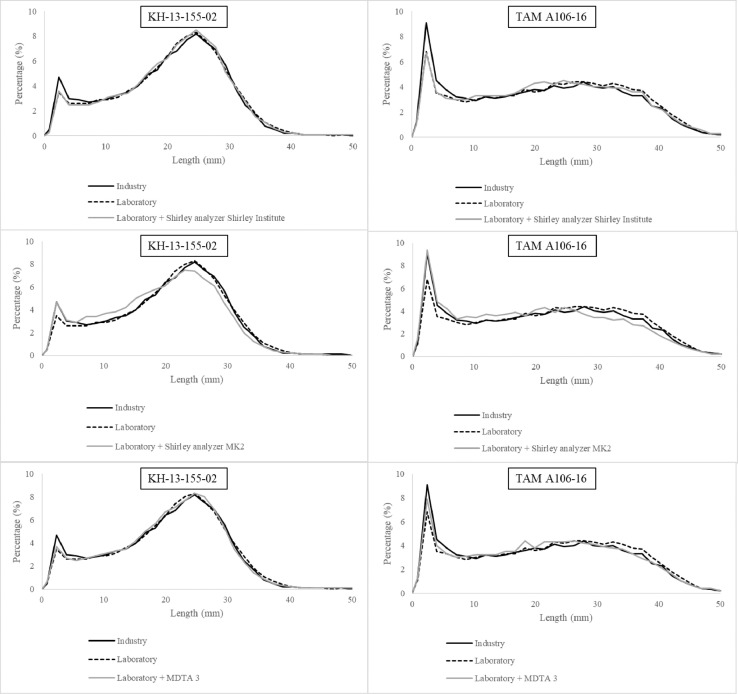
Fiber length distributions by number of two samples from industry-scale gin, laboratory-scale gin, laboratory-scale gin + Shirley analyzer from Shirley institute, laboratory-scale gin + Shirley analyzer MK2, and laboratory-scale gin + MDTA 3.

Fiber length parameters are extracted from distributions. The following examples are fiber length distributions of two genetic materials from the data set, KH-13-155-02 and TAM A106-16 (Fig. 2).

The Shirley analyzer from the Shirley Institute processes the samples and creates an intermediate distribution between industry- and laboratory-scale ginned lint types. The Shirley analyzer MK2 seems to have over-processed the samples and shortened the overall length of the fibers, increasing the differences in the distribution in comparison with industry-scale ginned lint. The MDTA-3 seems to simulate the industry-scale cotton fiber length profile better than the other tested machines.

## Conclusions

Industry-scale and laboratory-scale ginning have features that create differences in fiber length distributions. The differences can be minimized with additional processing for the laboratory-scale ginned lint. We tested the processing effect of three different instruments and verified that the MDTA 3 is the best instrument among the three tested to minimize length differences with industry-scale ginning. Any model of the MDTA system based on the same mechanical opening action should provide similar results.

## Declaration of Competing Interest

The authors declare that they have no known competing financial interests or personal relationships that could have appeared to influence the work reported in this paper.

## Appendix Supplementary materials

Image, application 1
