# Supplementary material for: A method to improve cotton fiber length measurement for laboratory analysis
Source: MethodsX. 2020 Mar 12;7:100859. doi: 10.1016/j.mex.2020.100859 (PMC7139154; doi:10.1016/j.mex.2020.100859)
Supplement: Supplementary file 1 [file mmc1.docx]

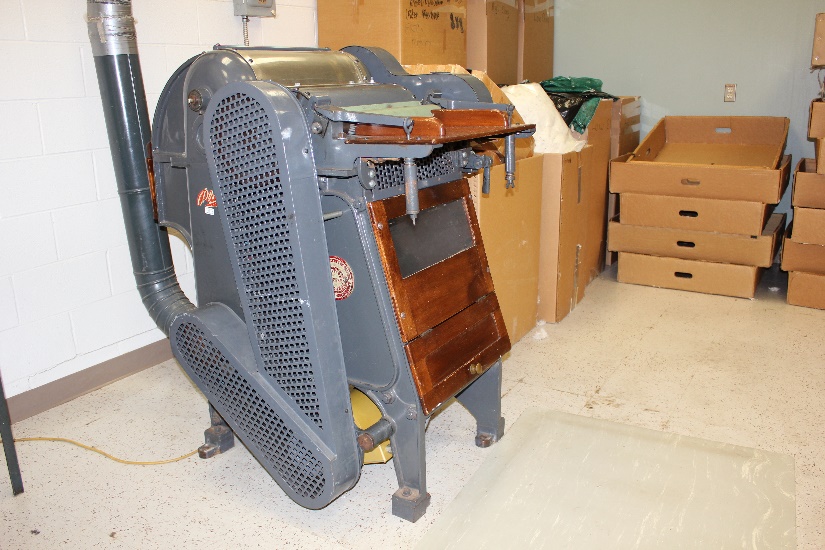


**A**

**B**

**C**


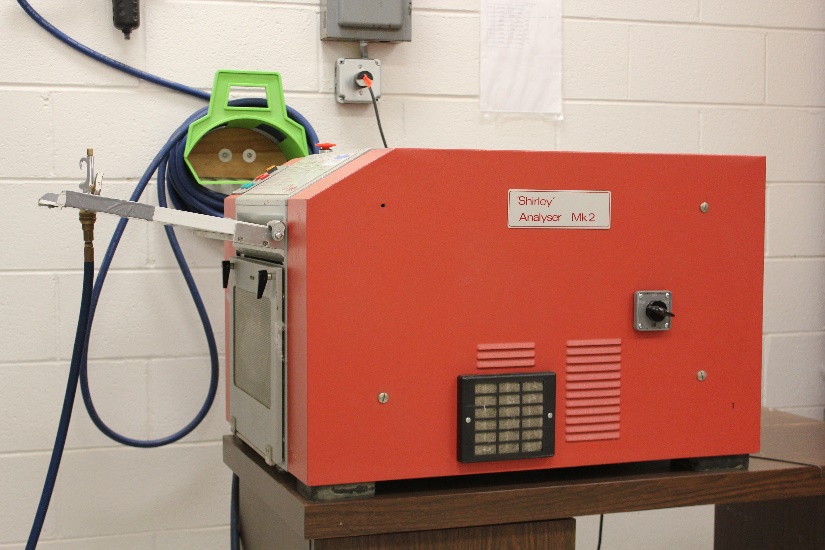


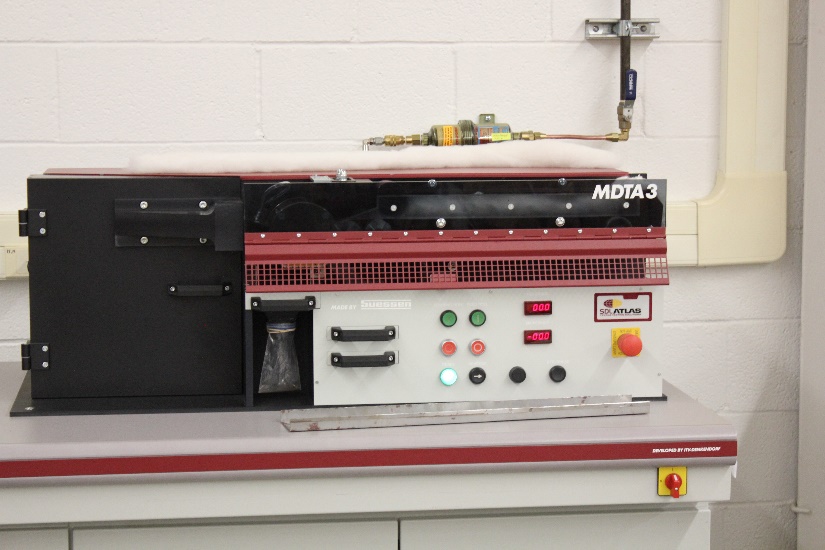


Figure S1. Laboratory-scale lint cleaners used in this study. Shirley analyzer from Shirley Institute (A), Shirley analyzer MK2 from SDL Atlas (B), MDTA-3 from Suessen (C).

Table S1. List of fiber length parameters from the dataset of genetic materials used in this experiment. Data obtained from industry-scale ginned lint.

| **Genetic Materials** | **Short fiber content by number (%)** | **Mean length by number (mm)** | **Average length of the 5% longer fibers (mm)** |
| --- | --- | --- | --- |
| Half and Half | 43.2 | 14.0 | 27.4 |
| KH-13-263-02 | 39.8 | 16.3 | 32.8 |
| PD-2 | 33.3 | 16.8 | 30.7 |
| TM-1 | 39.0 | 17.0 | 34.0 |
| Coker 100 wilt | 31.3 | 18.0 | 32.3 |
| Fox Big Boll | 30.4 | 18.0 | 32.0 |
| Arkot A306 | 28.6 | 18.5 | 32.0 |
| Delcot 277 | 29.9 | 18.5 | 33.3 |
| Hart | 26.7 | 18.8 | 33.0 |
| KH-13-205-04 | 32.6 | 19.1 | 38.4 |
| Coker 312 | 29.4 | 19.1 | 34.3 |
| TAM 11T-08 LSU/ESU | 31.9 | 19.1 | 35.8 |
| PHY 72 | 27.6 | 19.3 | 34.5 |
| TAM C66-16 | 32.3 | 19.6 | 37.6 |
| KH-13-167-01 | 22.9 | 19.6 | 31.8 |
| KH-13-178-04 | 22.3 | 19.8 | 31.8 |
| KH-13-155-02 | 22.9 | 20.1 | 32.5 |
| KH-13-399-02 | 24.4 | 20.8 | 35.8 |
| TAM A106-16 | 31.0 | 21.1 | 40.9 |
| KH-13-299-02 | 26.8 | 21.8 | 40.9 |
